# Incorporating Method Dissatisfaction into Unmet Need for Contraception: Implications for Measurement and Impact

**DOI:** 10.1111/sifp.12146

**Published:** 2021-02-17

**Authors:** Claire W. Rothschild, Win Brown, Alison L. Drake

**Affiliations:** ^1^ Claire W. Rothschild Department of Epidemiology University of Washington Seattle USA; ^2^ Win Brown The Bill & Melinda Gates Foundation Seattle WA USA; ^3^ Alison L. Drake Department of Global Health University of Washington Seattle USA

## Abstract

While unmet need for contraception is commonly used to assess programmatic needs, it inadequately captures the complexity of fertility and contraceptive preferences, including women's satisfaction with their contraceptive method. In their 2019 commentary, Sarah Rominski and Rob Stephenson propose reclassifying dissatisfied current users as having an unmet need for contraception. As revising the current definition based on their proposal would require significant investment to update survey and monitoring systems, understanding the potential impact on current estimates of unmet need is critical. We estimated the impact of this approach in a Kenyan cohort of modern contraceptive users. We found the prevalence of method dissatisfaction ranges from 6.6% (95% confidence interval [CI] 5.6–7.8%) to 18.9% (95% CI 17.1–20.9%); if applied nationally, this results in a large (approximately 25–70%) increase in Kenya's current estimate of unmet need for any contraception. Our findings suggest a large impact on unmet need estimates for equivalent populations. Overall, we advocate for better measurements of method satisfaction and acceptability, with metrics developed that are robust to socioeconomic gradients and validated in low‐ and middle‐income settings to ensure women's contraceptive needs are captured equitably.

Unmet need for contraception has been used to monitor reproductive health progress globally for more than half a century, when scholars first described gaps between women's stated contraceptive preferences and practices in the context of population programs focused on fertility (Westoff 1988). However, this metric creates challenges in tracking progress in the reproductive health agenda as it encompasses both fertility intentions and contraceptive use. For this reason, unmet need was deliberately avoided as a key performance monitoring indicator by the FP2020 Metrics Group (Brown et al. 2014). Today, global family planning (FP) initiatives such as FP2020 have selected alternate key indicators for monitoring progress, such as the modern contraceptive prevalence rate (mCPR) and additional modern contraceptive users (Brown et al. 2014). Yet measures of “need” persist, due to the utility of unmet need and measures of demand satisfied (derived in part from estimates of unmet need) to estimate the magnitude of need for contraceptive services and justify investments in family planning programs.

Since the 1994 International Conference on Population and Development in Cairo redirected our field from fertility control to a broader sexual and reproductive rights agenda, repeated calls have been made to refine the measure of unmet need to account for women's preferences and goals beyond pregnancy prevention. In their 2019 commentary (Rominski and Stephenson 2019), Sarah Rominski and Rob Stephenson advocate for revision of the current definition of unmet need, incorporating women's satisfaction with their current contraceptive method. The authors argue that women who report using contraception, but are dissatisfied with their method, should not be classified as having a “met need” for contraception. Revising country‐level estimates of unmet need based on their approach would require adding new measures of method satisfaction to nationally representative contraceptive surveying such as the Demographic and Health Survey (DHS) and Performance Monitoring and Accountability 2020 (PMA2020).

Since method satisfaction among current users is not routinely collected in low‐ and middle‐income countries (LMIC) (Inoue, Barratt, and Richters 2015), it is unclear whether such investments would result in meaningful differences in current estimates of unmet need. In many survey instruments (including the DHS) method dissatisfaction is captured only among women who have discontinued a method. As a result, method satisfaction is not holistically assessed for all contraceptive users and method dissatisfaction and discontinuation may be considered equivalent. A few studies in LMIC have reported high method satisfaction, particularly among long‐acting reversible (LARC) method users (Hubacher et al. 2015; Harrison and Goldenberg 2017). However, evidence suggests many women tolerate contraception to meet their fertility needs, despite its failure to meet their preferences (Severy and Newcomer 2005; Fathizadeh, Salemi, and Ehsanpour 2011; Schwarz et al. 2019).

Recent qualitative research in Burundi and the Democratic Republic of the Congo highlights that tolerability of side effects depends on both individual and contextual factors. These include relationship status and stability, strength of motivation for preventing pregnancy, contextual understandings of the socioeconomic and health effects of side effects, and pessimism about alternative contraceptive options (Schwarz et al. 2019). Other barriers, such as user fees and provider reluctance or refusal to remove LARC methods, may also contribute to dissatisfied, but continued, use (Newton and Hoggart 2015; Howett et al. 2019). These findings demonstrate the often difficult trade‐offs that woman are forced to make between their fertility and contraceptive preferences—trade‐offs not currently captured in metrics such as unmet need.

We used data from a prospective cohort study in Kenya to determine whether there are meaningfully different population‐level estimates of unmet need with the proposed new metric that incorporates method satisfaction. Women using modern, reversible forms of contraception were enrolled while attending public FP or maternal and child health clinics in Western Kenya (Festin et al. 2016). Additional details of participant eligibility, recruitment, and baseline characteristics of women in the analysis sample are available in the Supporting Information. Participants were asked to respond to short text message‐based surveys weekly for a period of 24 weeks after clinic attendance about their experiences with contraceptive use, including experience of side effects, method switching and discontinuation, and method satisfaction. In this analysis, we examined prevalence of method dissatisfaction among 990 women who rated their overall satisfaction with their current method at least once over follow‐up. Method satisfaction was measured on a 5‐point Likert scale, from “very dissatisfied” to “very satisfied.” We defined method dissatisfaction using two definitions: a narrow definition that included “very dissatisfied” or “dissatisfied” (versus “neutral” to “very satisfied”), and a broader definition that additionally included “neutral” as dissatisfied. While method “neutrality” is not equivalent to dissatisfaction, prior work in measurement of healthcare satisfaction suggests that the “satisfied” choice often represents average or adequate care (Collins and O'Cathain 2003; Poot et al. 2014); by extension, the “neutral” choice can be interpreted as a failure to meet this average standard, implying programmatic and clinical gaps in fully addressing women's contraceptive preferences. We estimated the mean weekly point prevalence of method dissatisfaction among participants who reported using modern contraception, averaged over 24 weeks. Log‐binomial generalized estimating equation models were used to account for repeated measures. Primary models were otherwise unadjusted.

Mean weekly prevalence of modern method dissatisfaction was 6.6% (95% confidence interval [CI] 5.6–7.8%) and 18.9% (95% CI 17.1–20.9%) using the narrow and broad definitions of method dissatisfaction, respectively. Dissatisfaction ranged from 7.7% (95% CI 6.4–9.3%) in the first 4 weeks to 4.9% (95% CI 3.9–6.3%) in weeks 21–24 of follow‐up using the narrow definition, and from 20.7% (95% CI 18.5–23.1%) to 17.1% (95% CI 15.0–19.4%) using the broad definition (Figure 1).

**FIGURE 1:**
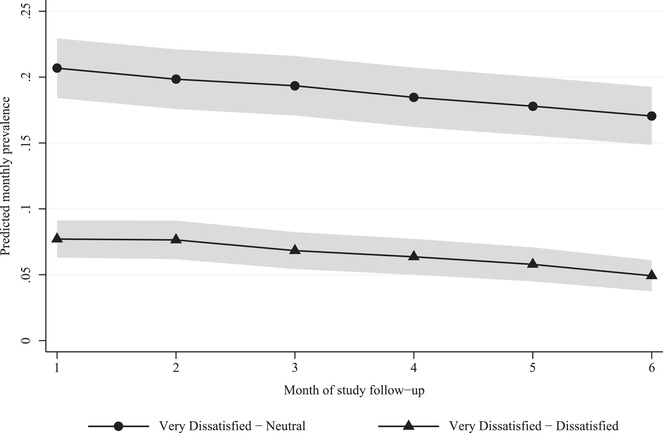
Prevalence of modern contraceptive method dissatisfaction NOTE: Mean monthly prevalence of modern method dissatisfaction was estimated using log‐binomial generalized estimating equations (GEE) with an exchangeable working correlation structure, robust standard errors, and adjusting for dummy variables of month of study follow‐up. 95% confidence intervals shown in the figure were estimated using the Delta method. Weekly observations were censored if missing or if no modern method use was reported that week. Month of follow up was defined using standard 4‐week intervals, with month 1 corresponding to weeks 0 through 4 poststudy enrollment, month 2 corresponding to weeks 5–8, and so forth.

Method dissatisfaction, when defined to include method “neutrality,” was substantial over the 24‐week period. Additional information on dissatisfaction by method and user type are provided in the Supporting Information. By both definitions, method dissatisfaction decreased modestly after 12 weeks postclinic attendance, to 64–82% of baseline levels (*p* < 0.05). These findings are consistent with several explanations: method acceptability may increase over time through increased method familiarity, resolution of side effects, or switch to an alternative method. Alternatively, since method satisfaction was assessed only among current contraceptive users, decreases in levels of dissatisfaction may be driven by a selection effect, in which the most dissatisfied women discontinue contraception altogether. Regardless, persistent method dissatisfaction at 6 months among women continuing contraception indicates that many women tolerate contraceptive methods ill‐suited to their needs and preferences.

To approximate impact on national estimates of unmet need, we multiplied the prevalence of method dissatisfaction by the mCPR among all women in Kenya (44.6%) (PMA2020), divided this quantity by the total population of Kenyan women of reproductive age, and added this proportion of dissatisfied users to the proportion with unmet need. We estimate that up to 1.2 million of the 6.1 million Kenyan women of reproductive age who use modern contraception (PMA2020) have some dissatisfaction with their method. If we applied Rominski and Stephenson's revised definition of unmet need based on method dissatisfaction in our sample to modern method users Kenya nationally,^1^ this would translate to an approximately 25% (using the narrow definition of dissatisfaction) to 70% (using the broad definition) increase in unmet need for any contraception (modern or traditional) from the current national estimate of 11.5% based on PMA data (Figure 2) (PMA2020).

**FIGURE 2:**
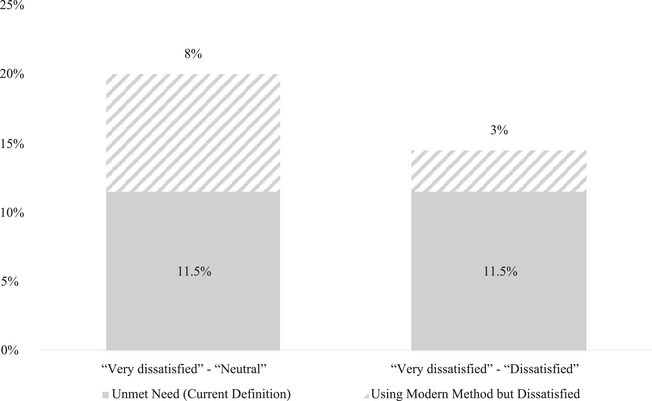
Potential magnitude of reclassifying Kenyan women dissatisfied with their current method as having “unmet need”NOTE: Projections are based on a total Kenyan female population of reproductive age of 13.7 million. Using PMA2020 Round 7 indicators for Kenya, we assume a modern contraceptive prevalence rate in this group of 44.6% and unmet need for any contraception (modern or traditional) of 11.5%. Number of current modern method users dissatisfied with their method was calculated by multiplying the total estimated population of modern method users (44.6% of 13.7 million women) by our estimated prevalence of method dissatisfaction (6.6% using the narrow and 18.9% using the broad definition). This produces estimates of approximately 400,000 dissatisfied (or 3% of the total reproductive age female population) using the narrow and 1.2 million dissatisfied (8%) using our broad definition of method dissatisfaction which would be added to the current 11.5% of women with unmet need. We estimate method dissatisfaction among modern contraceptive users only, which means that revised estimates of unmet need may be even higher if dissatisfaction among traditional method users were incorporated.

Our findings suggest that defining unmet need per Rominski and Stephenson's proposal could substantially increase estimates of unmet need, and investments in collecting data on method dissatisfaction among contraceptive users in LMIC may be warranted. However, decisions in how to measure and define method satisfaction matter. We observed large qualitative differences in levels of method dissatisfaction based on how we chose to define women whose satisfaction with their current method was “neutral.” Furthermore, interpretations of the concept of “satisfaction” may vary and this construct has not been well characterized among women in LMIC. In general healthcare settings, patient satisfaction is conceived as a result of individual expectations, which may be influenced by sociodemographic characteristics and prior experiences of care (Linder‐Pelz 1982; Conway and Willcocks 1997; Bowling, Rowe, and McKee 2013). Some studies have found reports of higher satisfaction among individuals with low socioeconomic status, including lower educational attainment and income and rural residence (Carlson et al. 2000; Al‐Rabeah et al. 2016); however, evidence is mixed and comparison across studies is difficult due to the lack of standard measures of satisfaction (Batbaatar et al. 2017). Any newly defined unmet need indicator would ideally ensure that all measurement components, including method satisfaction, are robust to socioeconomic gradients to ensure the needs of marginalized groups are captured and to prevent exacerbation of inequities. Efforts to develop measures of contraceptive method dissatisfaction that are stable across sociodemographic variables, and validated in LMIC with the highest burden of unmet need for contraception, are essential to adequately and equitably capture women's contraceptive needs and preferences.

While we concur with Rominski and Stephenson's appraisal that unmet need is a flawed indicator, and could be improved upon by incorporating satisfaction, their proposal to improve the metric is shortsighted. Developing population‐level contraceptive indicators that are rooted in principles of person‐centeredness and sexual and reproductive rights is critical to measure programmatic successes and gaps. Moreover, a new indicator would ideally reflect an individual's current needs, making it useful for both population‐level programming as well as individual‐level healthcare (Moreau et al. 2019). We believe it is necessary to critically evaluate all components reflected in the unmet need metric, including contraceptive acceptability and coercion, the fluid and dynamic nature of fertility intentions, and—perhaps most importantly—the problematic nature of defining “need” without asking women directly if they *want* to use contraception (Moreau et al. 2019).

Efforts to create a new metric of unmet need require rethinking a number of conventional measures. Standard assessment of fertility preferences and pregnancy intendedness, which are incorporated into the standard definition of unmet need, may fail to capture nuanced, unresolved, or ambivalent intentions, or their dynamism across the life course (Higgins 2017; Santelli et al. 2003; Schwarz et al. 2007; Schwarz et al. 2007). Simple dichotomization of women as having or not having “need” for contraception based on their fertility preferences remains problematic both for population‐level measurement and for individual‐level patient care. A recent study in Kenya in a cohort of FP clients found that over 40% reported ambivalent fertility intentions over a 2‐year period (Wekesa, Askew, and Abuya 2018). Formative research is crucial for designing metrics and programs that better address the specific reproductive health needs of this substantial proportion of contraceptive users.

Reformulation of an unmet need metric might also incorporate what Leigh Senderowicz terms “contraceptive autonomy” (Senderowicz 2020), a concept which combines informed choice and access to choice with women's own perceptions of contraceptive need. Senderowicz's work builds upon previous conceptualizations of contraceptive “acceptability” (Severy and Newcomer 2005) by emphasizing respect for women's autonomy as a prerequisite for met need. Similarly, Moreau et al. have proposed unmet demand as a distinct indicator from unmet need for contraception (Moreau et al. 2019). In sum, both Moreau and Senderowicz propose that need for contraception be defined based on women's own stated contraceptive desires or intentions, a radical departure from the current formulation.

Centering women's voices is critical for truly reimagining unmet need for contraception. This will require substantial formative work: new metrics should be developed through triangulation using qualitative methodology that allows women to describe contraceptive needs in their own words, survey research methods to develop a new classification system, followed by additional qualitative methods to measure accuracy of the new classification system. Similarly, attention should be paid to developing new indicators of demand for contraception: in particular, efforts to distinguish women's desire to use contraception from her planned intentions to do so may illuminate underappreciated challenges on the demand side (Garcia and Mann 2003; Fishbein and Yzer 2003). Testing metrics within demographic surveys, including those with prospective measurement capacity such as PMA2020, is one approach to promote assessment of the predictive validity of a new indicator over time in the context of dynamic pregnancy and contraceptive intentions.

Developing new measures for assessing contraceptive need remains a priority for the rights‐based reproductive health community. Ultimately, this will require re‐envisioning how we conceptualize and measure both fertility and contraceptive preferences, of which method satisfaction is just one component.

## ETHICS STATEMENT

The study was approved by the Ethical Review Committee of Maseno University (Kenya). While the study did not require approval from University of Washington's Human Subjects Division (HSD) due to its “not engaged” determination, this analysis was approved by UW's HSD. Participants provided written consent prior to study enrollment.

## CONFLICTS OF INTEREST

The authors declare no competing interests.

## Supporting information

Supporting information

## Data Availability

Data are available on request from the authors.

## Footnotes

1 Estimated impacts on current unmet need for any contraception (modern or traditional) are approximations based on the assumption that method dissatisfaction in our sample is similar to that of Kenyan modern method users nationally who could be defined as having unmet need for contraception. This assumption may be invalid for several reasons: first, our sample includes women who may be defined as having a met need for contraception even in the case of no contraceptive use (i.e., women <2 years postpartum); second, our facility‐based sample may not be nationally representative of individual sociodemographic and reproductive characteristics associated with method dissatisfaction.
