# Supplementary material for: Incorporating Method Dissatisfaction into Unmet Need for Contraception: Implications for Measurement and Impact
Source: Stud Fam Plann. 2021 Feb 17;52(1):95–102. doi: 10.1111/sifp.12146 (PMC8048066; doi:10.1111/sifp.12146)
Supplement: Supplementary file 1 — Supporting information [file SIFP-52-95-s001.docx]

**Incorporating Method Dissatisfaction into Unmet Need for Contraception:**

**Implications for Measurement and Impact**

Supplemental Material

**Participant recruitment and eligibility**

The full cohort is comprised of 1,212 women enrolled from February to May 2018 during attendance at a family planning or maternal and child health clinic within 10 public health facilities in Western Kenya. Eligible women were ≥18 years old (or an emancipated minor ≥14 years old), had daily access to a mobile phone with a Safaricom SIM card, the ability to read and respond to text messages themselves or with the help of a trusted person, and were using a modern, reversible form of contraception at the time of study enrollment. Modern methods were defined based on the classification developed by Festin et al. (Festin et al. 2016)

**Supplemental tables and figures**

| **TABLE T1 Baseline characteristics of participants included in the analytic sample** | | |
| --- | --- | --- |
|  | **Analysis Sample** | |
|  | n | n (%) or median (IQR) |
| Age (years) | 915 | 26 (23, 30) |
| Completed education (years) | 932 | 8 (4, 13) |
| Married (legal or presumed) | 923 | 750 (82) |
| Number of living children | 889 | 2 (1, 3) |
| Youngest child aged <6 months | 889 | 262 (29) |
| *Fertility intentions* |  |  |
| Unsure intention to have children or unsure of preferred timing | 897 | 182 (20) |
| Desires no future children |  | 184 (21) |
| Desires next pregnancy in <1 year |  | 41 (5) |
| Desires next pregnancy in 1-2 years |  | 79 (9) |
| Desires next pregnancy in >2 years |  | 411 (46) |
| Contraceptive method type used at study enrollment |  |  |
| Injectables | 894 | 358 (40) |
| Implant |  | 374 (42) |
| Intrauterine device^a^ |  | 48 (5) |
| Pills^b^ |  | 64 (7) |
| Other modern^c^ |  | 50 (6) |
| FP user type |  |  |
| Initiating contraception | 891 | 241 (27) |
| Switching from one method type to another |  | 110 (12) |
| Continuing method used in past month |  | 540 (61) |
| ^a^ Hormonal intrauterine systems are not widely available in Kenya's public health system; all women in this sample reported use of the non-hormonal IUD or were unsure of their method. Therefore, IUD users in our sample are likely all Cu-IUD users. ^b^ Pills include daily combined and progestin-only oral contraceptives. ^c^ Other modern methods include condoms, modern fertility-awareness based methods (LAM, Two Days Method, Standard Days Method), and emergency contraceptive pills; among fertility-awareness method users, eligibility was assessed at study enrollment. Over the course of the study follow-up, women who reported using a "natural method" in weekly surveys were defined as modern method users unless the method was clearly specified as traditional (such as withdrawal). Notes: The full analytic sample is comprised of N=990 participants with at least one complete week-level observation in which they were using a modern method of contraception and reported their method satisfaction. | | |

**FIGURE A1 Mean weekly method dissatisfaction, by contraceptive method and user type**

Panel A Method dissatisfaction by current contraceptive method type

Panel B Method dissatisfaction by FP user type at study enrollment

Notes: Predicted probabilities of mean weekly method dissatisfaction were estimated using log-binomial generalized estimating equations with robust standard errors and an exchangeable correlation structure to account for repeated observations. 95% confidence intervals estimated using the Delta method. Women not using a modern method that week were not included in that weekly sample, but may be included in other weeks. Models were adjusted for method type (panel A) or FP user type (panel B), but are otherwise unadjusted.

**FIGURE A2 Method dissatisfaction, by time since clinic attendance and method type**

Panel A Method dissatisfaction, defined as “very dissatisfied” or “dissatisfied”

Panel A Method dissatisfaction, defined as “very dissatisfied” or “dissatisfied”

Notes: Fitted probabilities of mean monthly method dissatisfaction were estimated using log-binomial generalized estimating equations with robust standard errors and an exchangeable correlation structure to account for repeated observations. 95% confidence intervals estimated using the Delta method. Women not using a modern method that week were not included in that weekly sample, but may be included in other weeks. Models include the following covariates: weekly method type, month since clinic attendance, and an interaction term between method type and study month. Months are defined using a standard 4-week period, with month 1 corresponding to weeks 0 through 4 post-study enrollment, etc.

**References**

Festin, M. P., J. Kiarie, J. Solo, J. Spieler, S. Malarcher, P. F. Van Look, and M. Temmerman. 2016. "Moving towards the goals of FP2020 - classifying contraceptives." *Contraception* 94 (4):289-94. doi: 10.1016/j.contraception.2016.05.015.
